# Crystal structure of *N*-[(2-hy­droxy­naphthalen-1-yl)(4-methyl­phen­yl)meth­yl]acetamide

**DOI:** 10.1107/S2056989015004661

**Published:** 2015-03-14

**Authors:** Sharanbasappa Khanapure, Gajanan Rashinkar, Tarulata Chhowala, Sumati Anthal, Rajni Kant

**Affiliations:** a Department of Chemistry, Shivaji University, Kolhapur 416 004, M.S., India; b VeerNarmad South Gujrat University, Surat 395 007, Gujrat, India; c X-ray Crystallography Laboratory, Post-Graduate Department of Physics & Electronics, University of Jammu, Jammu Tawi 180 006, India

**Keywords:** crystal structure, naphthalene, acetamide, π–π inter­actions, hydrogen bonding

## Abstract

In the title mol­ecule, C_20_H_19_NO_2_, the naphthalene ring system subtends a dihedral angle of 82.50 (7)° with the benzene ring and an intra­molecular N—H⋯O hydrogen bond closes an *S*(6) ring. In the crystal, mol­ecules are linked by O—H⋯O hydrogen bonds, which generate *C*(8) chains propagating in the [010] direction. The crystal structure also features weak π–π inter­actions [centroid–centroid separation = 3.7246 (10) Å].

## Related literature   

For background to *N*-(substituted phen­yl)acetamides, see: Schleiss *et al.* (2008 ▸). For further synthetic details, see: Shaterian *et al.* (2008 ▸). For related structures, see: Mosslemin *et al.* (2007 ▸); NizamMohideen *et al.* (2009 ▸).
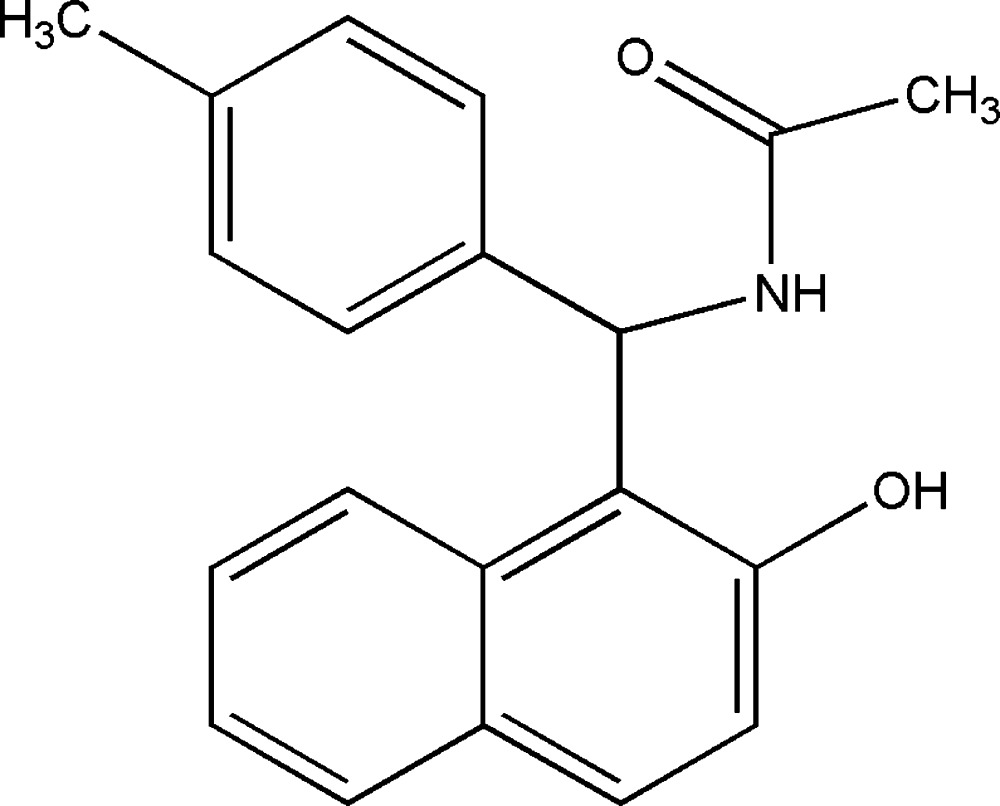


## Experimental   

### Crystal data  

C_20_H_19_NO_2_
*M* *_r_* = 305.36
Monoclinic, 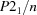

*a* = 10.4324 (4) Å
*b* = 14.0786 (5) Å
*c* = 11.0356 (4) Å
β = 98.741 (2)°
*V* = 1602.01 (10) Å^3^
*Z* = 4
Mo *K*α radiation
μ = 0.08 mm^−1^
*T* = 296 K
0.25 × 0.20 × 0.20 mm

### Data collection  

Bruker APEXII CCD diffractometer
Absorption correction: multi-scan (*SADABS*; Bruker, 2004 ▸) *T*_min_ = 0.980, *T*_max_ = 0.984
12272 measured reflections
2821 independent reflections
2391 reflections with *I* > 2σ(*I*)
*R*_int_ = 0.020

### Refinement  

*R*[*F* ^2^ > 2σ(*F* ^2^)] = 0.041
*wR*(*F* ^2^) = 0.122
*S* = 1.05
2821 reflections
209 parameters
H-atom parameters constrained
Δρ_max_ = 0.26 e Å^−3^
Δρ_min_ = −0.16 e Å^−3^

### 

Data collection: *APEX2* (Bruker, 2004 ▸); cell refinement: *SAINT* (Bruker, 2004 ▸); data reduction: *SAINT*; program(s) used to solve structure: *SHELXS97* (Sheldrick, 2008 ▸); program(s) used to refine structure: *SHELXL97* (Sheldrick, 2008 ▸); molecular graphics: *SHELXTL* (Sheldrick, 2008 ▸); software used to prepare material for publication: *SHELXTL*.

## Supplementary Material

Crystal structure: contains datablock(s) I, New_Global_Publ_Block. DOI: 10.1107/S2056989015004661/hb7375sup1.cif

Structure factors: contains datablock(s) I. DOI: 10.1107/S2056989015004661/hb7375Isup2.hkl

Supporting information file. DOI: 10.1107/S2056989015004661/hb7375Isup3.cml

**Fig. 1.**
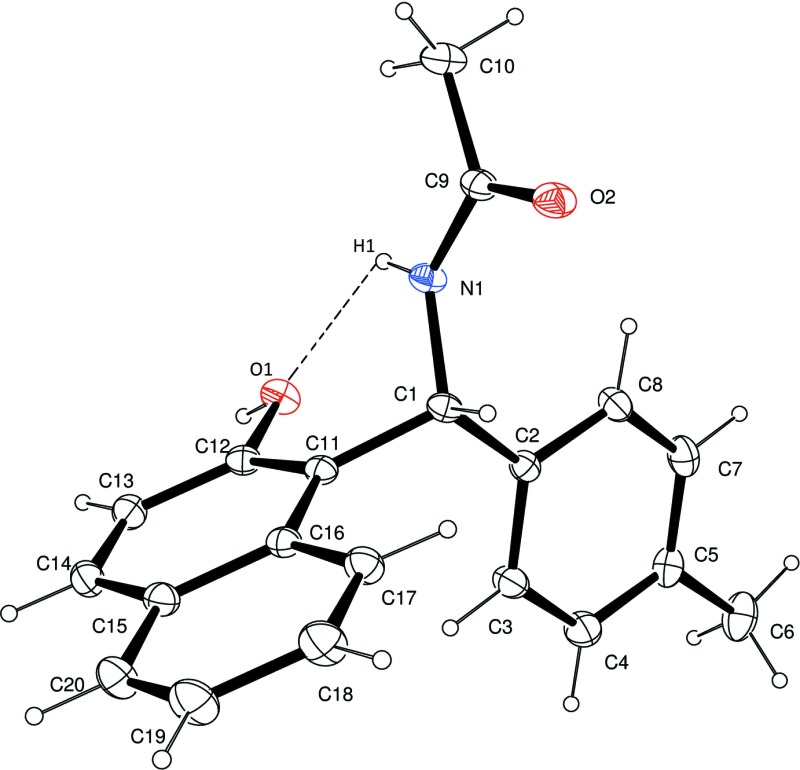
The molecular configuration of (I). Displacement ellipsoids are drawn at the 30% probability level.

**Fig. 2.**
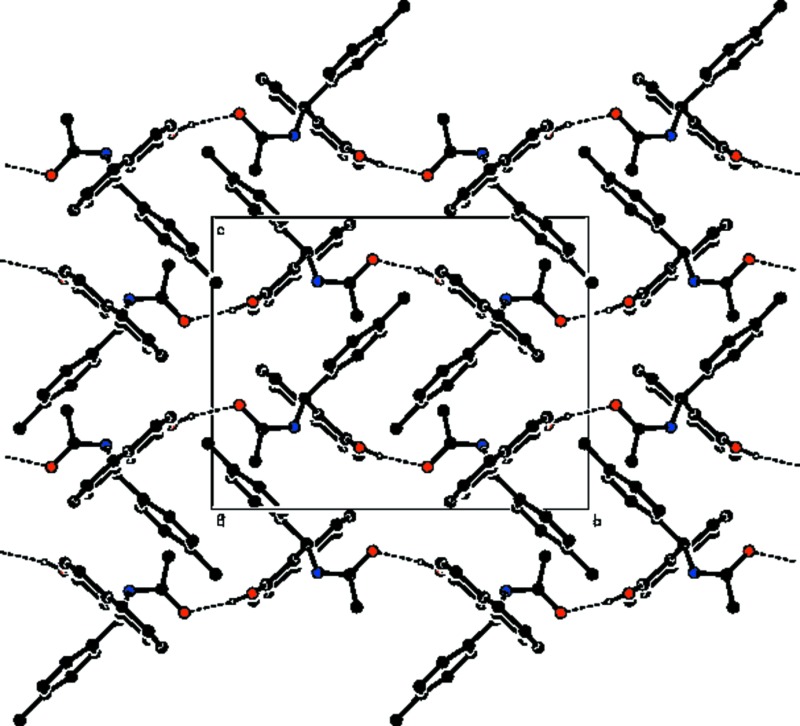
The packing arrangement of molecules viewed down the b axis.

. DOI: 10.1107/S2056989015004661/hb7375fig1.tif
The mol­ecular configuration of (I). Displacement ellipsoids are drawn at the 30% probability level.

b . DOI: 10.1107/S2056989015004661/hb7375fig2.tif
The packing arrangement of mol­ecules viewed down the *b* axis.

CCDC reference: 959797

Additional supporting information:  crystallographic information; 3D view; checkCIF report

## Figures and Tables

**Table 1:** Hydrogen-bond geometry (, )

*D*H*A*	*D*H	H*A*	*D* *A*	*D*H*A*
N1H1*A*O1	0.86	2.20	2.7396(16)	121
O1H1*B*O2^i^	0.82	1.85	2.6498(15)	165

Symmetry code: (i) 
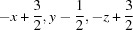
.

## supplementary crystallographic information

### S1. Comment

1-Amidoalkyl-2-naphthol scaffolds are of significant medicinal relevance since they can be converted into hypertensive and bradycardiac active 1-aminoalkyl-2-naphthols by amine hydrolysis reactions [Schleiss *et al.*, 2008]. As part of our studies in this area, we now describe the synthesis and structure of the title compound, (I).

The conformation of (I), together with the atom-numbering scheme, is shown in Fig. 1. In the structure, all bond lengths are comparable with those in previously reported structures (Mosslemin *et al.*, 2007, NizamMohideen *et al.*, 2009). Atom O1 deviating by 0.009 (1) Å from the least squares plane of the naphthalene ring. The dihedral angle between the naphthalene and benzene ring(C2/C3/C4/C5/C7/C8) is 82.5 (10)°. Examination of non bonded contacts reveals the presence of one N—H···O intramolecular hydrogen bond between N1 and hydroxyl atom O1 *via* H1 which results in the formation of pseudo six membered ring with S(6) graph-set motif. In this crystal, adjacent molecules are interconnected through O—H···O hydrogen bonds, which link the molecules into chains running along *b* axis. The crystal structure is further stabilized by π-π interactions between phenyl rings [centroid-centroid separation = 3.725 Å, interplaner spacing = 3.571 Å and centroid shift = 1.06 Å] where *Cg*1 and *Cg*2 represents the centre of gravity of rings (C2/C3/C4/C5/C7/C8) and (C11—C16), respectively.

### S2. Experimental

The compound *N*-[(phenyl)-(2-hydroxy-naphthalen-1-yl)- methyl]acetamide was synthesized by using benzaldehyde, 2-naphthol and acetamide by using Cp2ZrCl2 as a catalyst at room temperature. A mixture of 2-naphthol (1 mmol), benzaldehyde (1 mmol), acetamide (1.2 mmol) and zirconocene dichloride (20 mol%) was stirred in ethylene dichloride (5 ml) at room temperature for 10 h. After completion of reaction, as indicated by TLC, the reaction mixture was quenched in cold water. The obtained crude solid was filtered and purified by column chromatography on silica gel (Merck. 60–120 mesh, ethyl acetate: hexane)to afford the pure product in 72% yield. The identity of the compound was ascertained on the basis of FTIR, 1HNMR and 13CNMR spectroscopy as well as by mass spectrometry. The physical and spectroscopic data are consistent with the proposed structure and are in harmony with the literature values (Shaterian *et al.*, 2008). The IR spectrum exhibits broad absorption band at 3435 for O—H stretching and a sharp band at 3230 for N—H stretching of amide. The presence of amide group was apparent from strong absorptions at 1638 (C=O stretching) and 1597 (C—N stretching)·The 1H NMR (300 MHz, DMSO-d6) spectra of *N*-[(2-Hydroxynaphthalen-1-yl)(4-methylphenyl) methyl]acetamideexhibited singlets at δ 2.01 and 2.13 for protons of two methylgropus. The signals for amidic N—H and phenolic O—H protons appeared at 8.20 (*s*) and 9,96 (*s*) respectively. The two multiplets in the region 7.00–7.82 were assigned to ten aromatic protons and one methine proton. The proton decoupled 13 C NMR (75 MHz, DMSO-d6)spectra of *N*-[(2-Hydroxynaphthalen-1-yl) (4-methylphenyl)methyl]acetamidedisplay 19 distinct signals at 170.26, 153.43,139.67, 135.80, 132.64, 129.72, 129.08,128.86, 126.92,126.36, 123.53,122.98, 119.18, 118.82which is in agreement with the proposed structure. The Mass spectrum MS (EI): of this compound displayed the molecular ion peak at m/*z* = 306 (*M*+) which is in agreement with the proposed structure.

### S3. Refinement

All H atoms were positioned geometrically and were treated as riding on their parent C atoms, with C—H distances of 0.93–0.98 A; and with *U*_iso_(H) = 1.2*U*_eq_(C), except for the methyl groups where *U*_iso_(H) = 1.5*U*_eq_(C).

### Crystal data

**Table:**

C_20_H_19_NO_2_	*F*(000) = 648
*M**_r_* = 305.36	*D*_x_ = 1.266 Mg m^−^^3^*D*_m_ = 1.264 Mg m^−^^3^*D*_m_ measured by not measured
Monoclinic, *P*2_1_/*n*	Mo *K*α radiation, λ = 0.71073 Å
Hall symbol: -P 2yn	Cell parameters from 6588 reflections
*a* = 10.4324 (4) Å	θ = 2.5–28.2°
*b* = 14.0786 (5) Å	µ = 0.08 mm^−^^1^
*c* = 11.0356 (4) Å	*T* = 296 K
β = 98.741 (2)°	Block, colourless
*V* = 1602.01 (10) Å^3^	0.25 × 0.20 × 0.20 mm
*Z* = 4	

### Data collection

**Table:**

Bruker APEXII CCD diffractometer	2821 independent reflections
Radiation source: fine-focus sealed tube	2391 reflections with *I* > 2σ(*I*)
Graphite monochromator	*R*_int_ = 0.020
φ and ω scans	θ_max_ = 25.0°, θ_min_ = 2.4°
Absorption correction: multi-scan (*SADABS*; Bruker, 2004)	*h* = −12→12
*T*_min_ = 0.980, *T*_max_ = 0.984	*k* = −15→16
12272 measured reflections	*l* = −13→10

### Refinement

**Table:**

Refinement on *F*^2^	Primary atom site location: structure-invariant direct methods
Least-squares matrix: full	Secondary atom site location: difference Fourier map
*R*[*F*^2^ > 2σ(*F*^2^)] = 0.041	Hydrogen site location: inferred from neighbouring sites
*wR*(*F*^2^) = 0.122	H-atom parameters constrained
*S* = 1.05	*w* = 1/[σ^2^(*F*_o_^2^) + (0.0595*P*)^2^ + 0.5039*P*] where *P* = (*F*_o_^2^ + 2*F*_c_^2^)/3
2821 reflections	(Δ/σ)_max_ < 0.001
209 parameters	Δρ_max_ = 0.26 e Å^−^^3^
0 restraints	Δρ_min_ = −0.16 e Å^−^^3^

### Special details

**Table:**

Geometry. All s.u.'s (except the s.u. in the dihedral angle between two l.s. planes) are estimated using the full covariance matrix. The cell s.u.'s are taken into account individually in the estimation of s.u.'s in distances, angles and torsion angles; correlations between s.u.'s in cell parameters are only used when they are defined by crystal symmetry. An approximate (isotropic) treatment of cell s.u.'s is used for estimating s.u.'s involving l.s. planes.
Refinement. Refinement of *F*^2^ against ALL reflections. The weighted *R*-factor *wR* and goodness of fit *S* are based on *F*^2^, conventional *R*-factors *R* are based on *F*, with *F* set to zero for negative *F*^2^. The threshold expression of *F*^2^ > 2σ(*F*^2^) is used only for calculating *R*-factors(gt) *etc*. and is not relevant to the choice of reflections for refinement. *R*-factors based on *F*^2^ are statistically about twice as large as those based on *F*, and *R*- factors based on ALL data will be even larger.

### Fractional atomic coordinates and isotropic or equivalent isotropic displacement parameters (Å^2^)

**Table:**

	*x*	*y*	*z*	*U*_iso_*/*U*_eq_	
C1	0.70578 (14)	0.25289 (10)	0.87937 (14)	0.0381 (3)	
H1	0.6938	0.3100	0.9271	0.046*	
C2	0.78237 (14)	0.18214 (10)	0.96674 (14)	0.0379 (3)	
C3	0.72170 (16)	0.11312 (12)	1.02635 (16)	0.0510 (4)	
H3	0.6318	0.1079	1.0110	0.061*	
C4	0.79230 (18)	0.05162 (13)	1.10855 (18)	0.0598 (5)	
H4	0.7488	0.0058	1.1474	0.072*	
C5	0.92559 (18)	0.05652 (12)	1.13422 (16)	0.0527 (4)	
C6	1.0028 (2)	−0.01018 (15)	1.2240 (2)	0.0748 (6)	
H6A	1.0934	0.0045	1.2300	0.112*	
H6B	0.9881	−0.0744	1.1961	0.112*	
H6C	0.9761	−0.0032	1.3030	0.112*	
C7	0.98531 (17)	0.12598 (14)	1.07495 (17)	0.0578 (5)	
H7	1.0752	0.1312	1.0904	0.069*	
C8	0.91576 (16)	0.18805 (12)	0.99343 (16)	0.0508 (4)	
H8	0.9593	0.2346	0.9559	0.061*	
C9	0.83298 (15)	0.36630 (11)	0.77479 (15)	0.0426 (4)	
C10	0.89465 (19)	0.38493 (14)	0.66304 (18)	0.0614 (5)	
H10A	0.8856	0.3298	0.6112	0.092*	
H10B	0.9850	0.3988	0.6872	0.092*	
H10C	0.8529	0.4381	0.6191	0.092*	
C11	0.57167 (14)	0.21783 (10)	0.82499 (13)	0.0358 (3)	
C12	0.56126 (15)	0.14446 (10)	0.74112 (14)	0.0393 (4)	
C13	0.44045 (16)	0.10999 (11)	0.68525 (15)	0.0466 (4)	
H13	0.4361	0.0611	0.6280	0.056*	
C14	0.32992 (16)	0.14825 (12)	0.71506 (16)	0.0493 (4)	
H14	0.2501	0.1255	0.6772	0.059*	
C15	0.33371 (15)	0.22181 (12)	0.80244 (15)	0.0448 (4)	
C16	0.45625 (14)	0.25743 (10)	0.85831 (13)	0.0385 (4)	
C17	0.45539 (16)	0.33110 (12)	0.94630 (16)	0.0492 (4)	
H17	0.5338	0.3556	0.9851	0.059*	
C18	0.34242 (18)	0.36669 (15)	0.97517 (19)	0.0632 (5)	
H18	0.3450	0.4150	1.0330	0.076*	
C19	0.22255 (18)	0.33162 (16)	0.9191 (2)	0.0685 (6)	
H19	0.1459	0.3565	0.9391	0.082*	
C20	0.21922 (17)	0.26107 (14)	0.83527 (19)	0.0601 (5)	
H20	0.1393	0.2378	0.7983	0.072*	
N1	0.77811 (12)	0.28136 (9)	0.78136 (12)	0.0424 (3)	
H1A	0.7856	0.2408	0.7246	0.051*	
O1	0.67338 (11)	0.10791 (8)	0.71078 (11)	0.0497 (3)	
H1B	0.6595	0.0546	0.6818	0.075*	
O2	0.83171 (12)	0.42693 (8)	0.85572 (11)	0.0548 (3)	

### Atomic displacement parameters (Å^2^)

**Table:**

	*U*^11^	*U*^22^	*U*^33^	*U*^12^	*U*^13^	*U*^23^
C1	0.0395 (8)	0.0307 (7)	0.0462 (8)	−0.0022 (6)	0.0133 (7)	−0.0001 (6)
C2	0.0374 (8)	0.0347 (8)	0.0420 (8)	−0.0016 (6)	0.0073 (6)	−0.0045 (6)
C3	0.0395 (9)	0.0529 (10)	0.0601 (10)	−0.0034 (7)	0.0065 (8)	0.0127 (8)
C4	0.0596 (11)	0.0546 (11)	0.0628 (12)	−0.0048 (8)	0.0017 (9)	0.0175 (9)
C5	0.0580 (11)	0.0473 (10)	0.0486 (10)	0.0063 (8)	−0.0051 (8)	−0.0065 (7)
C6	0.0827 (15)	0.0659 (13)	0.0670 (13)	0.0196 (11)	−0.0172 (11)	−0.0003 (10)
C7	0.0391 (9)	0.0678 (12)	0.0630 (11)	0.0021 (8)	−0.0038 (8)	−0.0070 (9)
C8	0.0410 (9)	0.0523 (10)	0.0587 (10)	−0.0089 (7)	0.0063 (7)	−0.0005 (8)
C9	0.0406 (8)	0.0347 (8)	0.0531 (9)	−0.0017 (6)	0.0090 (7)	0.0078 (7)
C10	0.0683 (12)	0.0529 (10)	0.0681 (12)	−0.0112 (9)	0.0271 (10)	0.0078 (9)
C11	0.0382 (8)	0.0299 (7)	0.0399 (8)	−0.0006 (6)	0.0079 (6)	0.0058 (6)
C12	0.0444 (8)	0.0305 (7)	0.0443 (8)	0.0006 (6)	0.0108 (7)	0.0051 (6)
C13	0.0550 (10)	0.0365 (8)	0.0472 (9)	−0.0065 (7)	0.0045 (7)	−0.0016 (7)
C14	0.0449 (9)	0.0481 (9)	0.0527 (10)	−0.0073 (7)	0.0004 (7)	0.0039 (7)
C15	0.0400 (9)	0.0455 (9)	0.0493 (9)	−0.0001 (7)	0.0078 (7)	0.0082 (7)
C16	0.0405 (8)	0.0361 (8)	0.0400 (8)	−0.0001 (6)	0.0090 (6)	0.0063 (6)
C17	0.0449 (9)	0.0520 (10)	0.0528 (10)	−0.0019 (7)	0.0138 (7)	−0.0055 (8)
C18	0.0571 (11)	0.0668 (12)	0.0699 (12)	0.0024 (9)	0.0240 (9)	−0.0157 (10)
C19	0.0452 (10)	0.0796 (14)	0.0850 (14)	0.0078 (9)	0.0238 (10)	−0.0075 (11)
C20	0.0389 (9)	0.0704 (12)	0.0715 (12)	−0.0007 (8)	0.0103 (8)	−0.0005 (10)
N1	0.0474 (8)	0.0336 (7)	0.0491 (8)	−0.0052 (5)	0.0166 (6)	−0.0001 (5)
O1	0.0517 (7)	0.0348 (6)	0.0656 (8)	0.0005 (5)	0.0183 (6)	−0.0076 (5)
O2	0.0699 (8)	0.0352 (6)	0.0617 (8)	−0.0099 (5)	0.0179 (6)	0.0019 (5)

### Geometric parameters (Å, º)

**Table:**

C1—N1	1.4657 (19)	C10—H10B	0.9600
C1—C11	1.519 (2)	C10—H10C	0.9600
C1—C2	1.525 (2)	C11—C12	1.380 (2)
C1—H1	0.9800	C11—C16	1.425 (2)
C2—C3	1.380 (2)	C12—O1	1.3653 (18)
C2—C8	1.380 (2)	C12—C13	1.403 (2)
C3—C4	1.383 (2)	C13—C14	1.358 (2)
C3—H3	0.9300	C13—H13	0.9300
C4—C5	1.378 (3)	C14—C15	1.412 (2)
C4—H4	0.9300	C14—H14	0.9300
C5—C7	1.377 (3)	C15—C20	1.412 (2)
C5—C6	1.506 (3)	C15—C16	1.423 (2)
C6—H6A	0.9600	C16—C17	1.422 (2)
C6—H6B	0.9600	C17—C18	1.362 (2)
C6—H6C	0.9600	C17—H17	0.9300
C7—C8	1.379 (3)	C18—C19	1.398 (3)
C7—H7	0.9300	C18—H18	0.9300
C8—H8	0.9300	C19—C20	1.354 (3)
C9—O2	1.237 (2)	C19—H19	0.9300
C9—N1	1.3325 (19)	C20—H20	0.9300
C9—C10	1.498 (2)	N1—H1A	0.8600
C10—H10A	0.9600	O1—H1B	0.8200
			
N1—C1—C11	110.12 (12)	H10A—C10—H10C	109.5
N1—C1—C2	111.48 (12)	H10B—C10—H10C	109.5
C11—C1—C2	113.60 (11)	C12—C11—C16	118.85 (14)
N1—C1—H1	107.1	C12—C11—C1	118.83 (13)
C11—C1—H1	107.1	C16—C11—C1	122.32 (13)
C2—C1—H1	107.1	O1—C12—C11	117.61 (13)
C3—C2—C8	117.48 (15)	O1—C12—C13	120.52 (14)
C3—C2—C1	121.80 (13)	C11—C12—C13	121.84 (14)
C8—C2—C1	120.65 (14)	C14—C13—C12	119.71 (15)
C2—C3—C4	121.09 (16)	C14—C13—H13	120.1
C2—C3—H3	119.5	C12—C13—H13	120.1
C4—C3—H3	119.5	C13—C14—C15	121.33 (15)
C5—C4—C3	121.59 (17)	C13—C14—H14	119.3
C5—C4—H4	119.2	C15—C14—H14	119.3
C3—C4—H4	119.2	C14—C15—C20	121.69 (16)
C7—C5—C4	116.94 (16)	C14—C15—C16	118.98 (15)
C7—C5—C6	121.31 (18)	C20—C15—C16	119.33 (16)
C4—C5—C6	121.74 (19)	C17—C16—C15	117.02 (14)
C5—C6—H6A	109.5	C17—C16—C11	123.71 (14)
C5—C6—H6B	109.5	C15—C16—C11	119.26 (14)
H6A—C6—H6B	109.5	C18—C17—C16	121.57 (16)
C5—C6—H6C	109.5	C18—C17—H17	119.2
H6A—C6—H6C	109.5	C16—C17—H17	119.2
H6B—C6—H6C	109.5	C17—C18—C19	120.91 (18)
C5—C7—C8	121.93 (16)	C17—C18—H18	119.5
C5—C7—H7	119.0	C19—C18—H18	119.5
C8—C7—H7	119.0	C20—C19—C18	119.33 (17)
C7—C8—C2	120.96 (16)	C20—C19—H19	120.3
C7—C8—H8	119.5	C18—C19—H19	120.3
C2—C8—H8	119.5	C19—C20—C15	121.82 (18)
O2—C9—N1	121.89 (15)	C19—C20—H20	119.1
O2—C9—C10	121.78 (14)	C15—C20—H20	119.1
N1—C9—C10	116.33 (15)	C9—N1—C1	124.00 (13)
C9—C10—H10A	109.5	C9—N1—H1A	118.0
C9—C10—H10B	109.5	C1—N1—H1A	118.0
H10A—C10—H10B	109.5	C12—O1—H1B	109.5
C9—C10—H10C	109.5		
			
N1—C1—C2—C3	−148.79 (15)	C11—C12—C13—C14	−1.0 (2)
C11—C1—C2—C3	−23.7 (2)	C12—C13—C14—C15	−0.6 (2)
N1—C1—C2—C8	34.33 (19)	C13—C14—C15—C20	−179.08 (16)
C11—C1—C2—C8	159.45 (14)	C13—C14—C15—C16	1.2 (2)
C8—C2—C3—C4	−0.8 (3)	C14—C15—C16—C17	−179.63 (15)
C1—C2—C3—C4	−177.72 (16)	C20—C15—C16—C17	0.6 (2)
C2—C3—C4—C5	−0.1 (3)	C14—C15—C16—C11	−0.2 (2)
C3—C4—C5—C7	0.5 (3)	C20—C15—C16—C11	−179.93 (15)
C3—C4—C5—C6	179.83 (18)	C12—C11—C16—C17	178.05 (14)
C4—C5—C7—C8	−0.1 (3)	C1—C11—C16—C17	−1.4 (2)
C6—C5—C7—C8	−179.40 (18)	C12—C11—C16—C15	−1.3 (2)
C5—C7—C8—C2	−0.8 (3)	C1—C11—C16—C15	179.22 (13)
C3—C2—C8—C7	1.2 (2)	C15—C16—C17—C18	−0.6 (2)
C1—C2—C8—C7	178.18 (15)	C11—C16—C17—C18	−179.98 (16)
N1—C1—C11—C12	56.06 (17)	C16—C17—C18—C19	0.1 (3)
C2—C1—C11—C12	−69.78 (17)	C17—C18—C19—C20	0.2 (3)
N1—C1—C11—C16	−124.50 (14)	C18—C19—C20—C15	−0.2 (3)
C2—C1—C11—C16	109.65 (15)	C14—C15—C20—C19	179.99 (18)
C16—C11—C12—O1	179.95 (12)	C16—C15—C20—C19	−0.3 (3)
C1—C11—C12—O1	−0.6 (2)	O2—C9—N1—C1	3.4 (2)
C16—C11—C12—C13	2.0 (2)	C10—C9—N1—C1	−175.71 (14)
C1—C11—C12—C13	−178.56 (13)	C11—C1—N1—C9	125.81 (15)
O1—C12—C13—C14	−178.92 (14)	C2—C1—N1—C9	−107.16 (16)

### Hydrogen-bond geometry (Å, º)

**Table:**

*D*—H···*A*	*D*—H	H···*A*	*D*···*A*	*D*—H···*A*
N1—H1*A*···O1	0.86	2.20	2.7396 (16)	121
O1—H1*B*···O2^i^	0.82	1.85	2.6498 (15)	165

Symmetry code: (i) −*x*+3/2, *y*−1/2, −*z*+3/2.
