# Supplementary figures and images for: Crystal structure of N-[(2-hy­droxy­naphthalen-1-yl)(4-methyl­phen­yl)meth­yl]acetamide
Source: Acta Crystallogr E Crystallogr Commun. 2015 Mar 14;71(Pt 4):o235. doi: 10.1107/S2056989015004661 (PMC4438828; doi:10.1107/S2056989015004661)

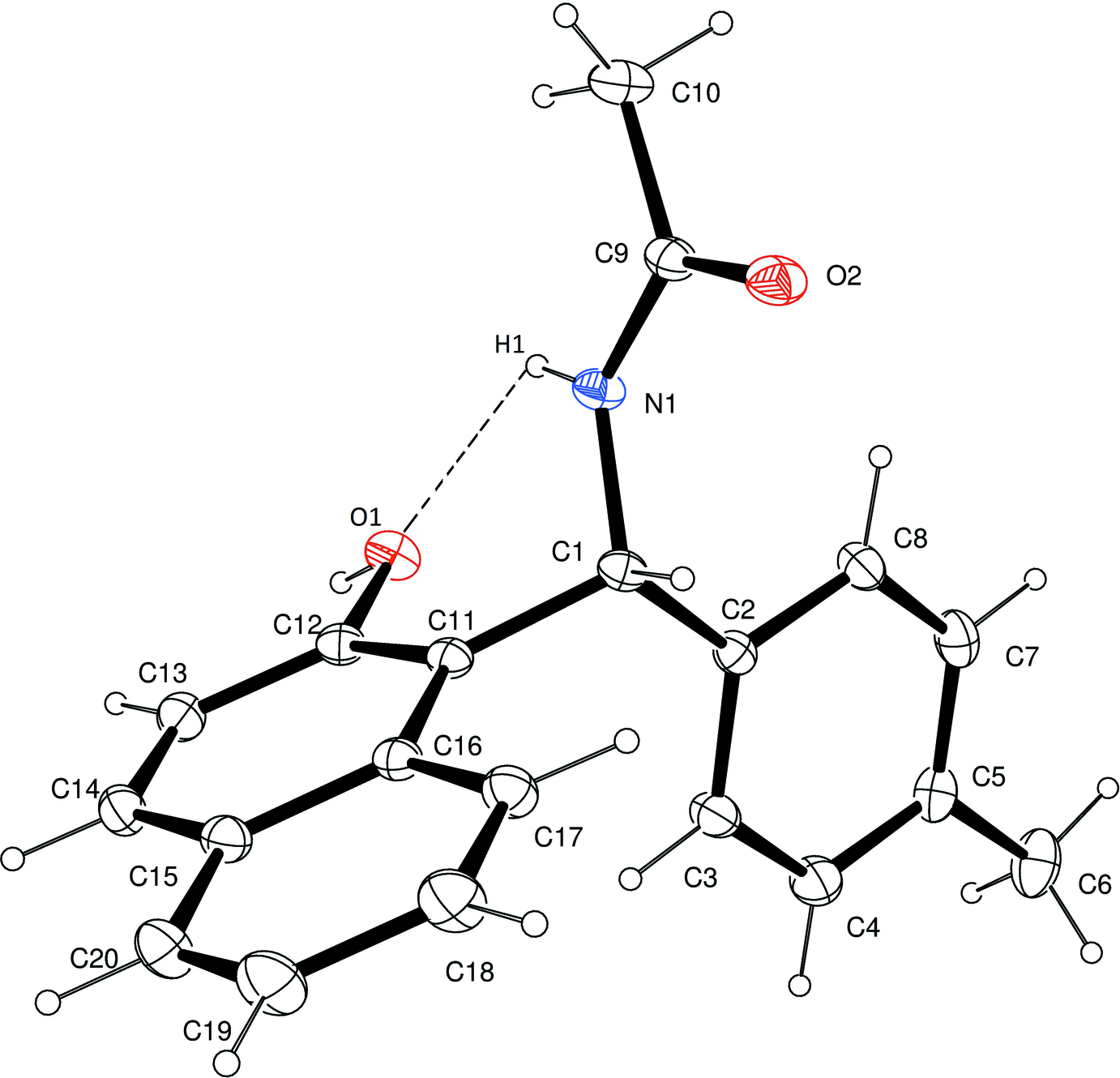

Supplement: Supplementary file 4 [file e-71-0o235-fig1.tif]

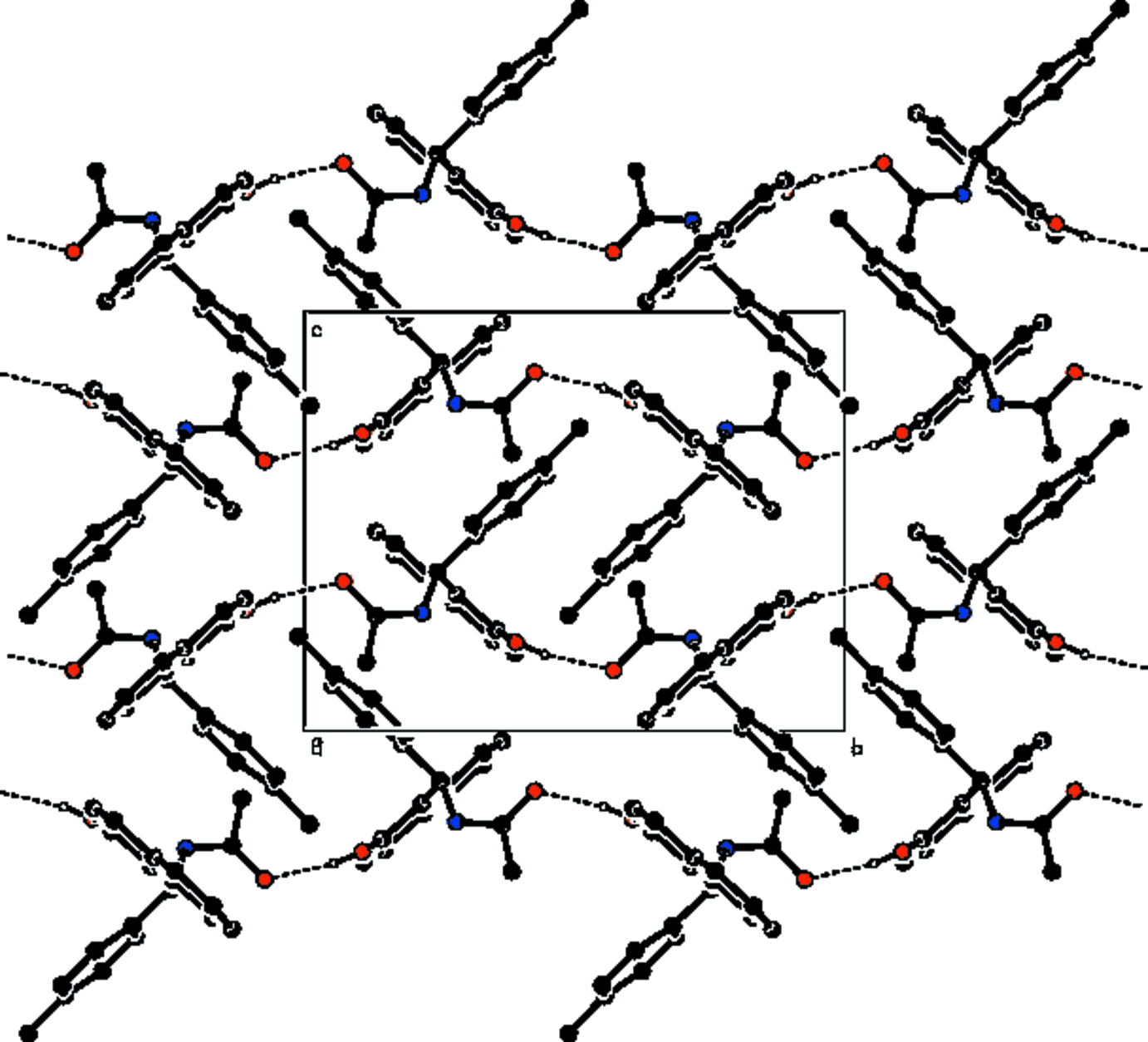

Supplement: Supplementary file 5 [file e-71-0o235-fig2.tif]
